# Antioxidant and anti-atherosclerotic potential of Banana (*Musa *spp): A review of biological mechanisms for prevention and protection against atherosclerosis

**DOI:** 10.22038/AJP.2022.20616

**Published:** 2023

**Authors:** Ferrari Carlos Kusano Bucalen

**Affiliations:** *Institute of Biological and Health Sciences (ICBS), Federal University of Mato Grosso (UFMT), Barra do Garças, Brazil*

**Keywords:** LDL, Triglyceride, Antioxidant, Lutein, Catechin, Quercetin

## Abstract

**Objective:**

To review the antioxidant and anti-atherosclerotic potential of whole banana, banana flour, and its bioactive compounds.

**Materials and Methods:**

A non-systematic review of the literature covering the past 20 years, using the following databases and searching bases: PUBMED/MEDLINE: https://www.ncbi.nlm.nih.gov/pubmed/; Google Scholar: https://scholar.google.com.br/; and “Literatura Latinoamericana em Ciências da Saúde”/Latin American Literature in Life Sciences [LILACS]: http://lilacs.bvsalud.org/, was done. Studies with incomplete methodology and design were excluded.

**Results:**

Bananas from different species are a good source of carbohydrates, dietary fiber, proteins, polyunsaturated fatty acids, potassium, carotenoids, flavonoids, vitamin C and E, phytosterols, gallocatechin, catechin, and other polyphenols. Some of these compounds play trigger important biological roles as antioxidants or anti-atherosclerotic and cardiovascular protective substances. This review summarizes and explains thirteen protective biological mechanisms of banana bioactive compounds and banana products.

**Conclusion:**

Including banana and its products in dietary menus, in food products and nutraceuticals should improve cardiovascular health of the populations.

## Introduction

Banana (*Musa *spp) is one of the most eaten foods around the world. Banana pulp is rich in starch, fiber, vitamins C and A, thiamine, riboflavin, phosphorus, potassium, calcium, and iron (Thanaraj and Terry, 2011). Banana peel also contains fiber, proteins, polyunsaturated fatty acids, potassium, carotenoids, vitamin C and E, gallocatechin, catechin, and other polyphenols (López and Montano, 2014). The most important banana plant species comprise *Musa paradisiaca*, *M. cavendish*, *M. acuminata*, *M. balbisiana* and *M. sapientum* (Rahman and Kabir, 2003; Thanaraj and Terry, 2011).

 Nowadays, physicians, researchers and the pharmaceutical industry are very interested in food phytochemicals for health promotion and disease control. Thus, the research has focused on studying possible antioxidant, anti-inflammatory, antihypertensive, diuretic, hepato-protective, cardio-protective, neuroprotective, hypolipidemic and hypoglycemic effects of plant extracts and foods (Bakour et al., 2021; Ferrari et al., 2016; Ferrari, 2020; Hoseini et al., 2021; Shirzad et al., 2021; Yarahmadi et al., 2021). 

 Due to increased pharmaceutical interest in natural products as a source of both antioxidants and anti-atherosclerotic compounds (Orekhov et al., 2013; Orekhov, 2013; Ferrari et al., 2016), especially bioactive compounds with capacity to inhibit low density lipoprotein (LDL) cholesterol oxidation and/or molecules capable to decrease cholesterol absorption and/or down-regulate liver cholesterol (Ferrari, 2013; Sobenin et al., 2013), the present study reviewed the roles of banana bioactive compounds in prevention and protection against atherosclerosis. 

## Materials and Methods

A non-systematic review of the literature covering the past 20 years (2000-2020), using the following databases and searching bases: PUBMED/MEDLINE: https://www.ncbi.nlm.nih.gov/pubmed/; Google Scholar: https://scholar.google.com.br/; and “Literatura Latinoamericana em Ciências da Saúde”/Latin American Literature in Life Sciences [LILACS]: http://lilacs.bvsalud.org/, was done. The major key words were “banana peel”, “banana pulp”, “banana products”, “antioxidant”, “antioxidant capacity”, “antioxidant activity”, “carotenoid”, “flavonoid”, “phenolics”, “cardiovascular prevention”, “cardiovascular disease”, “dyslipidemia” and “atherosclerosis”. Studies with incomplete methodology and design were excluded. Selected studies comprised theoretical as well as animal and laboratory studies and clinical trials.

## Results

**Reactive oxygen species and free radicals**

Reactive oxygen species comprise any chemical specie capable of independent existence exhibiting higher capacity to react with cell biomolecules, whereas free radicals are molecules with unpaired electrons in an orbital (Gutteridge and Halliwell, 2018; Patel et al., 2017).

The major source of free radicals is the respiratory mitochondrial chain, which releases the superoxide anion radical (O2-) that is converted by the enzyme superoxide dismutase (SOD) into hydrogen peroxide (H2O2), a highly reactive oxygen species (Gutteridge, 1995). Since H2O2 is a very reactive oxygen species, capable to generate the most damaging hydroxyl radicals (OH°), two antioxidant enzymes, catalase (CAT) and glutathione peroxidase (GPx), can metabolize this substance to innocuous water and atomic oxygen (Gutteridge, 1995; Lobo et al., 2010). Other free radicals comprise lipoperoxyl radicals, formed by lipid peroxidation, and peroxynitrite formed by reaction of the nitric oxide radical (NO°) with superoxide anion. Other reactive species include molecules from oxygen, nitrogen and chlorine, such as hypochlorous acid (HOCl-), singlet oxygen (1O2), peroxyl radical (HO2°), and ozone (Esterbauer, 1993; Ferrari et al., 2009; Gutteridge, 1995; Gutteridge and Halliwell, 2018; Kanner, 1994; Patel et al., 2017).

Free radicals, ROS and reactive nitrogen species (RNS) can cause lipid peroxidation of membrane phospholipids and of other cell lipids and they can oxidize proteins, enzymes, carbohydrates and nucleic acids (Ayala et al., 2014; Caimi et al., 2019; Gutteridge and Halliwell, 2018; Patel et al., 2017).

**Reactive oxygen, nitrogen and chlorine species: role in atherosclerosis**

The seminal paper that consolidated the cholesterol hypothesis of atherosclerosis was published by Steinberg et al. (1989). This paper emphasized the role of free radicals in the lipid peroxidation of blood lipids, especially the LDL cholesterol particles that, once oxidized (ox-LDL), are promptly engulfed by activated macrophages into the subendothelial space. Macrophages with intracytoplasmic accumulation of ox-LDL and other lipids become foam cells (Tertov et al., 1989). With the death of foam cells, atherosclerotic plaque is formed (Ferrari, 1998) through many specific molecular mechanisms reviewed by specialists (Chistiakov et al., 2017). The same study demonstrated that other cells from vascular walls (endothelial cells and vascular smooth muscle cells) can engulf cholesterol and other lipids, becoming foam cells (Chistiakov et al., 2017). It is important to observe that hyperlipidemia also induces neutrophil infiltration into the vascular wall, which can contribute to the genesis of the atherosclerotic plaque (Dreschler et al., 2010).

**Antioxidant activity of banana**

Due to their composition, bananas are recognized as a significant source of antioxidant activities *in vitro* and *in vivo*.

***In vitro*** **antioxidant activity of bananas**

Many banana species have presented considerable antioxidant activity by scavenging of ROS and RNS, as well as inhibiting lipid peroxidation reactions.

Baldi et al. (2012) reported that an alcoholic extract of banana had high antioxidant activity and considerable hydrogen peroxide-scavenging capacity. It is important to note that H_2_O_2_ induced atherogenic cascades and amplified inflammatory signaling, worsening evolution and stability of atherosclerotic plaque (Rajagopalan et al., 1996; Park and Oh, 2011).

Studying *Musa cavendish*, it was observed that gallocatechin concentration was higher in banana peel (158 mg/100 gr dry weight) than pulp (29.6 mg/100 gr dry weight) and that the antioxidant activity of banana peel was higher compared to the pulp (Someya et al., 2002).

Extracts of *M. cavendish*, imported by Japan, presented consistent antioxidant activity as measured by thiocyanate method, β-carotene bleaching assay, and the DPPH assay (Mokbel and Hashinaga, 2005).

Studying *Musa *spp in India**,** authors reported that banana extracts had potent antioxidant capacity, an effect due to its phenolic/flavonoid content (Darsini et al., 2012).

Other research showed that banana extracts had antioxidant activity that was responsible to decrease lipid peroxidation and scavenge H_2_O_2_, NO^°^, and O_2_^-^ (Kandasamy and Aradhya, 2014).

Bananas from Thailand also presented significant antioxidant activity measured by two different methods. In the ferric reducing antioxidant potential (FRAP) method, ripe samples had highest antioxidant activity compared to mature green and overripe samples, whereas when using DPPH assay ripe samples had lowest antioxidant activity compared to mature green and overripe group (Youryon and Supapvanich, 2017). In the same study, ripe and overripe had higher total phenolic content, whereas mature green had the higher flavonoid content (Youryon and Supapvanich, 2017).

Ripe crude bananas from Philippines, imported by Oman, had total phenolic content ranging from 131.72 mg of gallic acid equivalent (GAE)/100 g to 386.22 mg GAE/100 g whereas the total flavonoid content ranging from 0.16 µg/quercetin equivalent (QE)/g of dry powder to 8.51µg of QE/g of dry powder (Al Amri and Hossain, 2018). The same authors reported that banana had higher antioxidant activity measured by the DPPH assay.

In this respect, it is important to note that banana flower and stem or pseudostem are rich in fiber and polyphenolics with significant antioxidant activity (China et al., 2011; Bhaskar et al., 2012).

Total phenolic content is associated with high antioxidant capacity of a banana industrial by-product from Malaysia (Toh et al., 2016).

***In vivo*** **antioxidant activity of bananas**

Rats feeding banana extracts had decreased levels of both primary (hydroperoxides and conjugated dienes) and secondary (malondialdehyde (MDA)) lipid peroxidation products, whereas they had increased levels of antioxidant defense enzymes, such as SOD, CAT, and glutathione (GSH) (Vijayakumar et al., 2008).

Mice fed with banana extract had decreased lipid peroxidation measured by MDA content and increased GSH content (Baldi et al., 2012).

Due to their antioxidant content as well as the levels of carotenoids, phenolic 

compounds, vitamins, microelements, fiber and other bioactive compounds, regular eating of bananas and their products can afford many health benefits as listed in Table 1.

**Table 1:** Health benefits of bananas

**Antioxidant activity**	
Banana extracts are capable to scavenge both reactive oxygen species (H_2_O_2_) and oxygen & nitrogen free radicals (O_2_^-^; NO^°^) Antioxidant activity due to carotenoids, flavonoids, polyphenolics, dopamine, etc. Mice or rat feeding banana extracts had decreased markers of lipid peroxidation and increased values of CAT, GSH, and SOD	Baldi (2012) Kandasamy and Aradhya (2014) Darsini et al. (2012) Kulma and Szopa (2007) Aquino et al. (2018) Vijayakumar et al. (2008)
**Source of micronutrients**	
Banana pulp: starch, fiber, thiamine, riboflavin, vitamin C, vitamin A, iron, calcium, and potassium. It has also small amounts of copper, manganese, zinc and vitamin D Banana peel: starch, proteins, polyunsaturated fatty acids, vitamin C, vitamin E, potassium, carotenoids, and polyphenolics	Thanaraj and Terry (2011) López and Montano (2014)
**Anti-thrombotic activity**	
Crude banana inhibits platelet aggregation	Kandasamy et al. (2016)
**Anti-diabetic activity**	
Banana induced hypoglycemic effects in humans	Cressey et al., 2014
**Anti-atherosclerosis activity**	
Banana extracts and compounds effectively scavenge ROS and RNS Banana compounds protect endothelial against toxic and ROS/RNS damage Banana compounds decrease inflammation, including downregulation of endothelial cell adhesion molecules Banana compounds induces vasodilation and decreases blood pressure Banana extracts and compounds decrease blood cholesterol, triglycerides and improve HDL cholesterol	Kandasamy and Aradhya (2014) Ahmad et al. (2019) Izar et al. (2018) Krishnan and Vijayalakshmi (2005)

**Banana as a rich source of bioactive compounds with antioxidant activity**

Banana peel and pulp are rich in starch, total carotenoid, and antioxidant enzymes (CAT, peroxidase and ascorbate peroxidase) (Arora et al., 2008). In the same study, β-carotene content varied from 28 µg/100 g to 117.2 µg/100 g in pulps and from 49 µg/100 g to 241.91 µg/100 g in peel (Arora et al., 2008).

Another study reported a concentration of β-carotene from 45 µg/100 g to 7124 µg/100 g in banana cultivars from Solomon Islands (Englberger et al., 2010).

Banana flour, rich in resistant starch, phenolics, β-carotene and total carotenoids, presented also higher antioxidant activity by the DPPH method, by hydroxyl radical scavenging method and inhibition of lipid peroxidation assay (Moongngarm et al., 2014). Anyasi et al. (2018) reported that banana flour contains substantial amounts of organic acids (ascorbic acid, citric acid, and lactic acid), potassium and many polyphenolics, especially myricetin-glycosides.

Regarding carotenoid content, the following carotenoids were isolated from 36 banana varieties in China: violaxanthin, neoxanthin, antheraxanthin, α-cryptoxanthin, β-cryptoxanthin, lutein, α-carotene and β-carotene (Heng et al., 2017). Studying 15 Brazilian banana varieties, authors observed the occurrence of vitamin A, α-carotene, β-carotene, and lutein (Aquino et al., 2018).

It is also interesting to note that bananas are a source of lycopene. A study from Thailand reported that lycopene content of bananas represented 29.8% of the total carotenoid content observed in tomatoes Suwanaruang, 2016).

Banana (*M. cavendish*) is also rich in dopamine, with 80-560 mg/100 g in the peel and 2.5-10 mg in the pulp (Kanazawa and Sakakibara, 2000). In the same study, authors found that dopamine had high antioxidant activity, similar to gallocatechin gallate and ascorbic acid, but superior to the antioxidant activity presented by GSH, quercetin, luteolin and catechin. 

Other authors emphasize that due to the higher content of L-dopa and dopamine, banana, banana peel and its products should be useful to the treatment of Parkinson’s disease patients (Pereira and Maraschin, 2015). Another study also corroborated that *M. acuminata* and *M. sapientum* had high levels of dopamine in the pulp (Kulma and Szopa, 2007). Dopamine isolated from bananas was capable to improve LDL resistance to oxidation (Sidhu and Zafar, 2018).

**Banana bioactive compounds and atheroprotection**

Evidence from many biomedical studies as well as epidemiologic and clinical research showed that carotenoids, flavonoids, phenolics, tocopherols, and phytosterols, usually found in the different banana species, can display different antioxidant, anti-inflammatory and anti-atherosclerotic mechanisms, listed in Table 2 (Babu et al., 2012; Nguyen et al., 2017; Rebello et al., 2014; Sidhu and Zafar, 2018; Tsamo et al., 2015).

In addition to several health benefits, bananas have different compounds with protective effects against cardiovascular diseases.

**Table 2:** Banana lignins, carotenoids, flavonoids, and phenolics with anti-atherosclerotic and cardioprotective effects

Chemical specie	References
Carotenoids α-carotene, β-carotene and lutein violaxanthin, neoxanthin, antheroxanthin, α-criptoxanthin, β-criptoxanthin, lutein, α-carotene and β-carotene	Aquino et al. (2018) Heng et al. (2017)
Dopamine phenolic compound with two hydroxyl and one amino group	Kanazawa and Sakakibara (2000) Kulma and Szopa (2007) Pereira and Maraschin (2015)
Flavonoids and their glycosides catechins, proanthocyanins, anthocyanins, flavones, flavonols, and their glycosides Anthocyanins and its glycosides: delphinidin, pelargonidin, peonidin, and malvidin Gentisic acid, catechin, protocatechuic acid, caffeic acid, ferulic acid, cinnamic acid Chlorogenic acid, quercetin, naringenin Prodelphinidins, flavonol glycosides with quercetin-based chemical structures, procyanidin and flavonols Caffeic acid-hexoside, ferulic acid-hexoside and dihexoside, myricetin-hexoside, ferulic acid, sinapic acid, quercetin-hexoside, methylmyrecetin-hexoside, rutin, kaempferol-hexoside, kaempferol-rutinoside, isorhametin-rutinoside p-hydroxybenzoic acid, gallic and ferulic acid Lignins and vitamins C and B	Pereira and Maraschin (2015) Ahmad et al. (2019) Kandasamy and Aradhya (2014) Babu et al. (2012) Rebello et al. (2014) Tsamo et al. (2015) Nguyen et al. (2017) Ahmad et al. (2019)

**Lutein and atherosclerosis**

Evidence from epidemiologic, *in vitro* and *in vivo* studies indicated that lutein intake or supplementation can substantially reduce the risk of atherosclerosis by inhibiting both LDL deposits in arterial macrophages and atherosclerotic plaque progression (Dwyer et al., 2001).

It had been suggested that lutein as well as food carotenoids can inhibit macrophage uptake of oxidized LDL particles by decreasing cholesterol oxidation and reducing inflammation in the vascular domains (Howard and Thurnham, 2017).

Lutein can also decrease disease progression in patients with early atherosclerosis, since it reduces serum LDL, triglycerides, interleukin-6 (IL-6), and macrophage chemo-attractant protein-1 (Xu et al., 2013).

Studying 40 early atherosclerosis patients without clinical cardiac events, authors reported that serum lutein was inversely associated with IL-6 and positively associated with IFN-γ, whereas zeaxanthin was related to expression of vascular adhesion molecules and ApoE, and lycopene were negatively correlated with vascular adhesion molecules and LDL (Xu et al., 2012).

Wang et al. (2013) observed that lutein supplementation improved total antioxidant capacity of plasma and decreased both MDA and C-reactive protein in non-smoking healthy people.

Lutein is swallowed by blood mononuclear cells and it is capable to inhibit both gene expression and secretion of IL-6, IL-1β, and tumor necrosis factor (TNF) in stable coronary artery disease patients (Chung et al., 2017).

Another two mechanisms through which lutein affords protection against atherosclerosis comprise PPAR expression and inhibition of NADPH oxidase (Han et al., 2015).

**Lycopene and atherosclerosis**

In a randomized clinical control trial, supplementation with lutein significantly decreased carotid artery intima-media thickness, whereas the degree of atherosclerosis inhibition was greater among those who received lutein plus lycopene supplementation (Zhou et al., 2014).

 Daily supplementation with lycopene (5 mg/kg) during 4 weeks decreased by half the blood total cholesterol and the LDL cholesterol in rabbits (Lorenz et al., 2012). In the same study, lycopene did not improve aortic intima-media thickness or endothelial-dependent and independent aortic and carotid vasodilation.

A meta-analysis of clinical studies reported that a daily dosage of 25 mg of lycopene effectively reduced total blood cholesterol, accounting for a 10% reduction on the LDL cholesterol (Ried and Fakler, 2011).

Lycopene can reduce carotid-intima thickness, LDL, LDL oxidation, vascular adhesion molecules, vascular inflammatory cytokines, proliferation of smooth muscle cells, and endothelial damage (Palozza et al., 2010; Riccioni et al., 2008; Rissanen et al., 2002; Xu et al., 2012).

**Anti-atherosclerotic actions of catechins and quercetin**

O_2_^-^, H_2_O_2_ and HOCl^-^ can be scavenged by catechin and quercetin (Binsack et al., 2001; Pannala et al., 1997). This explains why dietary intake of polyphenolic-rich foods lower peroxynitrite formation, improve endothelial function, and decrease both LDL oxidation and atherogenesis (Stein et al., 1999; Waddingtol et al., 2004).

In this context, quercetin can enhance HDL cholesterol levels and reduce triglycerides levels, whereas it can diminish both blood pressure and LDL oxidation by ROS (Egert et al., 2009; Pfeuffer et al., 2013).

Catechin is responsible to decrease the release of pro-inflammatory cytokines in the vasculature, to reduce vascular inflammation and atherosclerotic lesion size (Moss and Ramji, 2016).

Other dietary flavonols improved HDL levels and decreased total cholesterol levels in clinical trials (Moss and Ramji, 2016).

Catechin, epicatechin and quercetin reduce endothelial platelet activation though enhancement of nitric oxide levels, whereas other polyphenols can decrease mitochondrial H_2_O_2_ and the subsequent activation of phospholipase C and cicloxigenase-1, which also materially decrease thromboxane A2 synthesis and platelet aggregation (Ludovici et al., 2018). Dietary polyphenols also partially inhibit activation of NADPH-oxidase protecting the endothelium against excessive amounts of the powerful oxidizing peroxinitrite, contributing to decreased atherothrombotic damage and progression (Ludovici et al., 2018; Zhang and Tsao, 2016).

These results corroborate previous finding that quercetin and catechin, two banana polyphenols, inhibited phosphokinase C dependent activation of NADPH-oxidase, decreasing the production of oxygen and nitrogen free radicals and platelet aggregation (Pignatelli et al., 2006). Quercetin and catechin are also responsible for increasing the nitric oxide production (Pignatelli et al., 2006), which is known to have important endothelial vasodilator effects (Ignarro et al., 2002).

Banana compounds such as gallocatechin gallate as well as campesterol and stigmasterol reduce cholesterol absorption, whereas dopamine and catechin improve resistance of LDL to oxidation, and quercetin, ferulic acid and catechins have arterial vasodilatory effects (Sidhu and Zafar, 2018).

Catechin and epicatechin also decreased the expression of vascular endothelial adhesion molecules and e-selectin, but sustainably increased endothelial-nitric oxide synthase and nitric oxide vascular releasing (Carnevale et al., 2014).

Catechin, epicatechin and other two polyphenolics were able to decrease the expression of inflammatory cytokines (IL-1β, TNF-α, and IL-6), to inhibit NFκB, to induce the expression of the nuclear releasing factor-2 (Nrf-2), to substantially decrease both the production of nitric oxide and peroxynitrite (by inhibition of nitric oxide synthase) and the amount of oxygen free radicals due to inhibition of myeloperoxidase and direct free radical-scavenging mechanisms (Marinovic et al., 2015).

**Banana phytosterols, tocopherols and chlorogenic acid as anti-atherosclerotic compounds**

Bananas of *M. acuminata*, *M. balbisiana* and *M. cavendish* are rich in β-sitosterol, ω-3 and ω-6 fatty acids, and contain substantial amounts of α-tocopherol (Vilela et al., 2014).

Tocopherols can scavenge ROS protecting LDL from oxidation and decreasing inflammation on the endothelial surfaces what was associated with reduced risk of atherosclerosis (Ferrari and Torres, 2003; Ferrari, 2004; Yang et al., 2009).

Also found in bananas, ß-sitosterol can potently lower inflammation, fat accumulation and decreased atherosclerosis, lowering both plasma and liver cholesterol (Kurano et al., 2018; Lei et al., 2017).

Chlorogenic acid, found in coffee and bananas, inhibited the oxidative enzymes cycloxigenase-1 and inducible form of nitric oxide synthase (iNOS), and partially suppressed the release of inflammatory cytokines (IL-1β, TNF-α, and IL-6), effects in part mediated by the NFκB (Hwang et al., 2014).

A literature review pointed out that chlorogenic acid has potential benefits against atherosclerosis because it decreases endothelial inflammatory biomarkers, thromboxane A2 synthesis and oxidative/nitrosative stresses, and it improves blood pressure and endothelial functions (Tajik et al., 2017).

***In vivo*** **studies with banana feeding and atherosclerotic markers evaluation**

Rats feeding a flavonoid rich extract from banana (*M. paradisiaca*) had decreased liver and heart levels of triglycerides, phospholipids, free fatty acids, cholesterol and MDA (Krishnan and Vijayalakshmi, 2005).

After administration of indomethacin rats presented increased levels of oxidative stress as measured by increased levels of lipid peroxidation (MDA content) and decreased glutathione content. In the same study, after feeding rats with *Musa sapientum* extracts there was an increase in glutathione and HDL values as well as a reduction in MDA, total cholesterol, triglycerides and LDL levels (Akinlolu et al., 2013).

In a Nigerian study, Wistar rats were separated in groups with a control, a hypercholesterolemic control, and subgroups feeding different banana species. It was found that banana (*M. sapientum*) peel extract completely restored the HDL levels and partially recovered total cholesterol, triglycerides and LDL levels towards the normal levels (Edenta et al., 2014).

Using a rat model of liver failure, in comparison to the control groups, rats eating banana peel extracts had improved levels of total cholesterol, triglycerides, LDL and VLDL cholesterol (Mosa and Khalil, 2015).

In Wistar rats submitted to a cholesterol rich diet, feeding a methanolic sterol-rich extract from banana (*M. sapientum*) partially restored total cholesterol, LDL, VLDL, and HDL, decreasing MDA and increasing SOD, CAT, and GSH (Dikshit et al., 2016).

Dietary intake of both flower and banana inflorescence from *M. balbisiana* Colla reversed deleterious effect of diabetes on blood biochemical markers, since there were decreases in total cholesterol, triglycerides, LDL and MDA, and enhancement on HDL and CAT serum concentrations (Borah and Das, 2017).

Extracts of *M. cavendish* and *M. acuminata* had a significant hypoglycemic effect in Wistar rats, and antioxidant capacity *in vitro* as measured by scavenging of DPPH radical, ferric reducing power, and hydrogen peroxide assay (Navghare and Dhawale, 2017).

Since hyperglycemia is a risk factor for atherosclerosis^, ^(Gaudreault et al., 2013), it is important to note that hypoglycemic effect of bananas can contribute to decreased coronary artery disease risk.

Considering some functional properties of banana, Kandasamy et al. (2016) found that banana crude extracts inhibited platelet aggregation. 

Since atherosclerosis origin and progression depend on inflammatory leukocyte recruiting and macrophage activation, banana peel compounds were found to inhibit both the expression of NFkB and chemoattractant and adhesion molecules; they were also associated with enhanced production of endothelial isoform of nitric oxide synthase (e-NOS), favoring vasodilation (Ahmad et al., 2019).

**Human clinical studies with banana feeding and atherosclerotic markers evaluation**

A clinical study with hypercholesterolemic subjects revealed only a small beneficial effect of eating bananas on glycemic and LDL/HDL ratio (Cressey et al., 2014).

In a randomized controlled clinical trial, the experimental group received nutritional counseling and 40 g of green banana biomass, whereas the control group received only a diet. After six months, there was a decrease in body mass index, fasting glucose, and systolic blood pressure only in the experimental group, whereas in both groups there were a decrement in waist and hip circumferences, fat mass (%), and fat intake (Izar et al., 2018).

More clinical studies are needed in order to evaluate possible anti-atherosclerotic benefits of eating bananas.

## Discussion

Based on research previously described regarding banana bioactive compounds, the suggested anti-atherosclerotic biological mechanisms are listed in Table 3.

**Figure 1:**
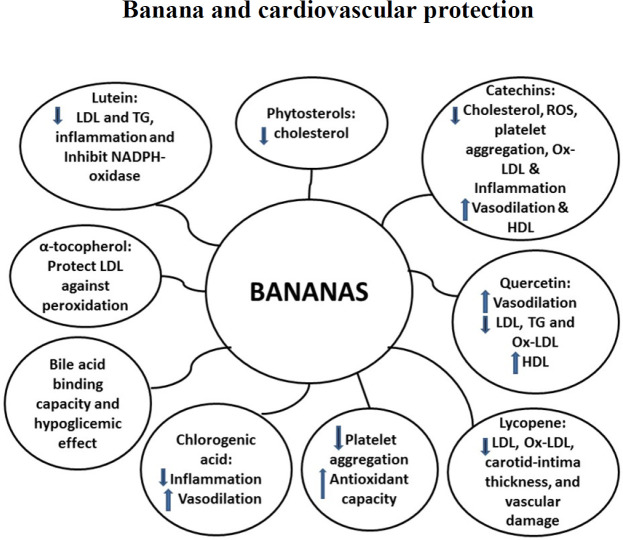
Anti-atherosclerotic mechanisms of banana bioactive compounds

Considering the major protective antioxidant, anti-atherosclerotic and cardiovascular mechanisms, Figure 1 summarizes the roles of banana bioactive compounds.

It is important to note that until today, there is lower quantity of experimental animal studies and only a few clinical studies that evaluated possible anti-atherosclerotic effects of banana supplementation. More research on antioxidant and cardioprotective effects is necessary considering the different banana species, varieties, postharvest conditions, and the diverse banana food preparations and products. Knowledge of the mechanisms suggested here can stimulate more research on the antioxidant and cardiovascular protective mechanism of banana and its bioactive compounds.

**Table 3:** Anti-atherosclerotic biological mechanisms of banana bioactive compounds

Banana extract or component/dose	Mechanism	References
Banana dry matter	The in vitro bile acid binding capacity of banana extracts was higher than 6 different fruit extracts	(Kahlon and Smith, 2007)
Six month randomized controlled trial comparing nutritional counseling *plus *40g of green banana biomass/day with placebo (control)	Eating green banana decreased body mass index, fasting blood glucose, and systolic blood pressure in humans	(Izar et al., 2018)
Dietary intake of a flavonoid rich fraction from banana	Decreasing lipid levels from heart and liver as well lipid peroxidation	(Krishnan and Vijayalakshmi, 2005)
Flower and Inflorescence stalk ethanolic extract at 250 mg/kg/day p.o of *Musa balbisiana Colla.*	Both improved blood lipid profile (total cholesterol, tryglycerides, and LDL), decreasing lipid peroxidation and enhancing HDL in rats	(Borah and Das, 2017)
5, 10 and 20 mg/kg body weight of *Musa sapientum*	Improved GSH and CAT levels and decreased SOD levels. It also decreased total cholesterol, tryglycerides and LDL and increased HDL levels in rats	(Akinlolu et al., 2013)
Feeding a methanolic extract of *Musa sapientum* (10, 20 and 40 mg/kg/day)	At 20 or 40 mg/kg doses the hypercholesterolemic effects were partially reversed. Banana extract improved total cholesterol and its fractions, decreased lipid peroxidation and restored the antioxidant enzymes (SOD and CAT) in rats	(Dikshit et al., 2016)
Ingesting aqueous peel extracts of the 3 different cultivars of *Musa sapientum* (100 mg/Kg bw)	Decreased both tryglycerides and total cholesterol in rats	(Edenta et al., 2014)
Eating banana peel extracts	Diminished total cholesterol, triglycerides, LDL and VLDL cholesterol in rats	(Mosa and Khalil, 2015)
Banana crude extracts	Inhibition of platelet aggregation as well as antifungal and antibacterial effects	(Kandasamy et al., 2016)
Eating banana 250 or 500 gr/day for 4 weeks	Decreased the mean glycemic values by 10% in humans	(Cressey et al., 2014)
Ethanolic extracts of *M. Cavendish* (250, 500 and 1000 mg/kg, p.o) and *M. acuminata* (100, 200 and 400 mg/kg, p.o)	They had significant hypoglycemic and antioxidant effects rats	(Navghare and Dhawale, 2017)
Banana bioactive compounds, such as catechin, dopamine, lutein, lycopene, quercetin, and tocopherols	Protect LDL against peroxidation	(Egert et al., 2009) (Howard and Thurnham, 2017). (Palozza et al., 2010) (Sidhu and Zafar, 2018) (Yang et al., 2009)

## Conflicts of interest

The authors have declared that there is no conflict of interest.
